# Differences in quality of life and cognition between the elderly and the very elderly hemodialysis patients

**DOI:** 10.1590/2175-8239-JBN-2018-0167

**Published:** 2019-03-18

**Authors:** Fernanda Siqueira Viana, Yolanda Eliza M. Boechat, Jocemir Ronaldo Lugon, Jorge Paulo Strogoff de Matos

**Affiliations:** 1 Universidade Federal Fluminense Faculdade de Medicina NiteróiRJ Brasil Universidade Federal Fluminense, Faculdade de Medicina, Niterói, RJ, Brasil.; 2 Universidade Federal Fluminense Faculdade de Medicina Departamento de Medicina Clínica NiteróiRJ Brasil Universidade Federal Fluminense, Faculdade de Medicina, Departamento de Medicina Clínica, Niterói, RJ, Brasil.

**Keywords:** Hemodialysis, Elderly, Very old, Cognition, Quality of life.

## Abstract

**Introduction:**

In the last decades, there was an expressive increase in the number of elderly patients with chronic kidney disease starting hemodialysis. Thus, our goal was to evaluate the profile of the elderly in chronic hemodialysis and to compare the cognition and quality of life of the younger elderly with those of the very elderly.

**Methods:**

Patients on hemodialysis for at least 3 months, who were 65 years of age or older when they started dialysis were invited to participate, and stratified according to age (under or over 80 years). The participants answered a clinical-epidemiological questionnaire and underwent cognitive tests (Mini Mental State Exam [MMSE], clock drawing test [CDT] and verbal fluency test [VFT]) and a quality of life assessment 36- Item Short Form Health Survey).

**Results:**

Of the 125 eligible patients, 124 agreed to participate. The mean age was 76 ± 6 years (28% ≥ 80 years), 56% were men and 55% had ≥ 8 years of schooling. Depression was suggested in 38%. The prevalence of cognitive deficit was 38%, 70% and 30%, by MEEM, CDT and VFT, respectively. The prevalence of any deficit was higher among the very elderly (94% vs. 72%, *p* = 0.007). Quality of life scores were similar between the two age groups, except for the functional capacity domain, worse in the group with ≥ 80 years (*p* = 0.033).

**Conclusion:**

Elderly patients on chronic hemodialysis have a high prevalence of cognitive deficits, especially the very elderly, but this group does not have a worse quality of life, except for functional capacity.

## Introduction

The aging of the world population and the consequent epidemiological transition in recent decades have caused a marked increase in the prevalence of elderly patients with end stage renal disease (ESRD), especially the oldest old or very old (≥ 80 years), treated with or without renal replacement therapy (RRT). In the United States, the incidence of hemodialysis (HD) among the elderly has increased 2.2-fold between 1996 and 2015.^1^ Epidemiological data is scarce in Brazil, but it is known that among the more than 120,000 dialysis patients in the country in 2016, about 11% were aged 75 or over.^2^

Despite the epidemiological relevance, there are still many gaps regarding the conditions of the elderly patients in dialysis treatment. Although the incidence of octogenarian and nonagenarian patients on dialysis has increased considerably in the last decades, the survival of this group remains modest.^3^^,^^4^ We still do not know to what extent prolonged survival may be followed by losses and limitations. However, we still don`t know whether there is any difference in the profile of the very old and of the elderly and those younger than 80 years of age in the chronic HD program.

Our study aims to assess the quality of life and cognition of the elderly in a chronic HD program, comparing patients under 80 years of age to those aged 80 years and over.

## Methods

### Study Design

This was a cross-sectional observational study, involving all four outpatient dialysis units in the city of Niterói, RJ (Clínica de Diálise do Ingá, Clínica de Doenças Renais São Lourenço, Clínica de Depuração Extra-Renal Ltda. e Clínica Nefrológica Ltda.), in the period from July 2016 to March 2017. All patients on chronic hemodialysis for at least 3 months and who had started treatment at 65 years or older were eligible to participate in the study, and those who underwent another type of RRT (peritoneal dialysis or renal transplantation) were taken off. The Research Ethics Committee of the Antônio Pedro University Hospital, Federal Fluminense University, Niterói, RJ approved this study, under CAAE number: 53503216.3.0000.5243.

### Assessments

After we obtained the informed consent, we assessed the clinical and epidemiological characteristics of the patients using questionnaires and the reviewed medical records. The laboratory profile was obtained by means of the chart, considering the most recent values at the time of entry into the study. The questionnaires were applied to the 15-item version of the geriatric depression scale (GDS)^5^ and the multidimensional health-related quality of life questionnaire - short form (SF)-36;^6^ both validated in Brazil.^7^^,^^8^ The GDS is an instrument of 15 items of dichotomized responses, for which 0 or 1 is scored, and these points are added to the final result.^5^ A value ≥ 6 was considered indicative of depression. The SF-36 encompasses eight domains: functional capacity, limitation by physical aspects, pain, general health, vitality, social aspects, limitations due to emotional aspects and mental health. It has no cut-off points; it is used to compare two or more populations, enabling comparisons also with the general population pattern. Scores are determined using the Likert method for summary evaluations, with linear transformation on a scale of 0 to 100. Larger scores indicate a better health-related quality of life.^9^

We performed the following tests for cognitive assessment: Mini Mental State Exam (MMSE),^10^ the clock drawing test (CDT)^11^ and the verbal fluency test (VFT) in the "animals" category. This category of VFT is part of the CERAD (Consortium to Establish a Registry for Alzheimer's Disease),^12^ a battery of neuropsychological tests widely used in Brazil and worldwide. All three have validation for our population.^13^^-^15

The cut-off points used to define the presence of cognitive deficits by the MMSE were 19 and 23, for patients with and without formal schooling, respectively.^16^ The CDT scores followed the instructions of Manos and Wu, with a cut-off point of 7/8 (case/no case) for a total of 10.^11^ The presence of cognitive impairment due to VFT was considered in the lists with less than 9 or 13 words, for individuals with less than 8 years of schooling or more than 8 years, respectively.^17^

### Statistical analysis

Continuous variables were expressed as mean ± standard deviation (SD) in cases of Gaussian distribution, and median with interquartile range (IQR) if non-Gaussian distribution; discrete variables were expressed by their frequency. Comparisons of the means between the groups were made by the *t*-test, when the distribution was Gaussian, or Mann-Whitney test, in case of non-Gaussian distribution. Comparisons between frequencies were made using the Fisher's exact test. We used logistic regression to analyze the variables associated with cognitive deficit. In all cases, the null hypothesis was rejected when the P value was less than 0.05. We ran the analyses using the SPSS program, version 18.0 for Windows (Chicago, Illinois, USA).

## Results

Of the 136 patients initially eligible to participate in the study, 11 were excluded for having started renal replacement therapy (RRT) by another method before they migrated to hemodialysis (7 per peritoneal dialysis and 4 per renal transplant). In addition, one refused to participate in the study and three did not perform the cognitive tests. A total of 124 patients were effectively included in the analysis.

### Clinical and demographic characteristics

The mean age at study entry was 76.0 ± 6.2 years, with 28.2% being 80 years or older at the time of the study, and of these, 37.1% initiated dialysis treatment with at least 80 years. Of the total number of patients evaluated, 55.6% were men, 58.1% were white, 54.8% had at least 8 years of schooling and 64.5% had a supplementary health plan. The main characteristics of the study population are described in Table 1. The very elderly group had more time on hemodialysis than the group of elderly individuals less than 80 years of age, with a median (IQR) value of 39 (16-73) months versus 21 (8 to 40) months, respectively; *p* = 0.0016. There was also a difference between the two groups in mean weight and body mass index (BMI), prevalence of diabetes, coronary artery disease, multiple morbidities, severe auditory deficit not corrected by prosthesis, thyroid disease and history of smoking (Table 1).

**Table 1:** Clinical and demographic characteristics

Variable	All (n= 124)	< 80 years (n= 89)	≥ 80 years (n= 35)	*p* value
Age (years)	76.0 ± 6.2	72.6 ± 3.8	82.9 ± 3.6	-
Male gender, n (%)	69 (55.6)	54 (60.7)	15 (42.9)	0.11
Whites, n (%)	72 (58.1)	54 (60.7)	18 (51.4)	0.42
Schooling ≥ 8 years, n (%)	68 (54.8)	51 (57.3)	16 (45.7)	0.32
Supplementary healthcare insurance, n (%)	80 (64.5)	56 (62.9)	24 (68.6)	0.68
Distance from home (km)	4 (2-11)	5 (2 - 12)	4 (2 - 10)	0.59
Age upon HD onset (years)	72.9 ± 5.8	70.5 ± 3.8	79.1 ± 5.2	-
Time in HD (months)	25 (11-58)	21 (8.5 - 40)	39 (16 - 73)	0.0016
Weight (kg)	65.6 ± 14.3	68.3 ± 14.2	59.4 ± 12.6	0.0016
BMI (kg/m^2^)	23.6 ± 5.2	24.4 ± 5.0	21.7 ± 5.1	0.0101
Diabetes, n (%)	70 (56.5)	56 (62.9)	14 (40.0)	0.0269
Arterial hypertension, n (%)	121 (97.6)	88 (98.9)	33 (94.3)	0.19
Coronary arterial disease, n (%)	38 (30.6)	33 (37.1)	5 (14.3)	0.0167
PAD, n (%)	22 (17.7)	19 (21.3)	3 (8.6)	0.12
Cerebrovascular disease, n (%)	29 (23.4)	22 (24.7)	7 (20.0)	0.64
Multiple morbidities, n (%)	50 (40.3)	41 (46.1)	9 (25.7)	0.0434
Severe auditory deficit, n (%)	19 (15.3)	8 (9.0)	11 (31.4)	0.0041
Severe visual deficit, n (%)	27 (21.8)	17 (19.1)	10 (28.6)	0.33
Thyroid disease, n (%)	22 (17.7)	10 (11.2)	12 (34.3)	0.0041
Amputation, n (%)	7 (5.6)	5 (5.6)	2 (5.7)	1.0
Smoking, n (%)	54 (43.5)	45 (50.6)	9 (25.7)	0.0155
Alcoholism, n (%)	12 (9.7)	10 (11.2)	2 (5.7)	0.51
Uncured cancer, n (%)	3 (2.4)	1 (1.1)	2 (5.7)	0.19
Recent hospital stay, n (%)	25 (20.2)	16 (18.0)	9 (25.7)	0.33

Values expressed by mean ± SD, median interquartile range or by frequency; HD = hemodialysis; peripheral arterial disease (PAD); Multiple morbidities = arterial hypertension or diabetes associated with the arterial disease manifests in more than one target-organ; recent interaction = last 3 months.

### Features associated with dialysis treatment

Of the 124 patients studied, 53.2% had DM as a cause of CKD, 66.1% initiated dialysis treatment at the hospital level and 63.7% had a double-lumen catheter, although 64.5% received previous follow-up by a nephrologist. Patients < 80 years of age were dialyzed for longer than those aged ≥ 80 years (*p* = 0.03). There was a tendency to have more patients with DM as a baseline disease in the group with < 80 years and a higher prevalence of long-stay tunnelled catheter in the group with ≥ 80 years. Table 2 depicts these and other characteristics associated with dialysis.

**Table 2:** Characteristics associated with the dialysis treatment, comparing patients < 80 years vs. ≥ 80 years

Variables	< 80 years (n = 89)	≥ 80 years (n = 35)	*p* value
HD frequency/week	3.56 ± 0.94	3.57 ± 1.01	0.96
3 x/week, n (%)	60 (67.4)	24 (68.6)	1.0
4 x/week, n (%)	15 (16.9)	6 (17.1)	1.0
5 or 6 x/week, n (%)	14 (15.7)	5 (14.3)	1.0
Time in HD/week (hours)	11.93±1.24	11.37±1.37	0.03
DM as baseline disease, n (%)	53 (59.6)	13 (37.1)	0.09
Hospital onset, n (%)	60 (67.4)	22 (62.9)	0.68
Prior follow up with a nephrologist, n (%)	59 (66.3)	27 (77.1)	0.28
Initial vascular access			
Native AVF, n (%)	27 (30.3)	11 (31.4)	1.0
Tunnelled catheter, n (%)	3 (3.4)	4 (11.4)	0.10
Temporary DLC, n (%)	59 (66.3)	20 (57.1)	0.41
Current vascular access			
Native AVF, n (%)	70 (78.7)	26 (74.3)	0.64
Tunnelled catheter, n (%)	11 (12.4)	7 (20.0)	0.27
Temporary DLC, n (%)	6 (6.7)	0 (0)	0.18
Vascular prosthesis, n (%)	2 (2.2)	2 (5.7)	0.32
Nº of prior catheters, n (%)			
0	20 (22.5)	7 (20.0)	1.0
≥ 3	24 (27.0)	13 (37.1)	0.28
Nº surgeries for AVF, n (%)			
0	3 (3.4)	3 (8.6)	0.35
≥ 3	13 (14.6)	12 (34.3)	0.02

Values expressed as mean ± SD or frequency; HD = hemodialysis; DM = diabetes *mellitus*; BMI = body mass index; PAD = peripheral arterial disease; DLC = double lumen catheter; AVF = arteriovenous fistula.

### Laboratory characteristics

There was no laboratorial difference in the comparison between patients < 80 years and those>= 80 years, except for serum triglycerides, which was higher among patients < 80 years; HDL and TSH was higher in patients aged ≥ 80 years. The percentage of patients immunized for hepatitis B was lower among those < 80 years (Table 3).

**Table 3:** Laboratorial characteristics, comparing patients < 80 years vs. ≥ 80 years

Variable	< 80 years (n = 89)	≥ 80 years (n = 35)	*p* value
Hemoglobin (g/dL)	10.6 ± 1.73	11.0 ± 2.17	0.28
Leucocytes (x 10^3^/mm^3^)	6.16 ± 2.24	6.14 ± 2.23	0.96
Platelets (x 103/mm^3^)	224.7 ± 98.75	219.3 ± 85.72	0.79
Pre-HD urea (mg/dL)	115.2 ± 35.12	110.1 ± 36.02	0.47
Post-HD urea (mg/dL)	35.17 ± 18.4	35.39 ± 20.0	0.95
Std Kt/V urea	2.41 ± 0.61	2.47 ± 0.66	0.64
Creatinine (mg/dL)	7.18 ± 2.3	6.48 ± 2.1	0.15
Potassium (mEq/L)	5.45 ± 0.94	5.34 ± 0.97	0.58
Calcium (mg/dL)	9.16 ± 0.84	9.37 ± 0.60	0.18
Phosphorus (mg/dL)	4.75 ± 1.20	4.69 ± 1.16	0.78
Glucose (mg/dL)	135.7 ± 69.88	141.9 ± 68.49	0.69
HbA1c (%)	6.35 ± 1.20	6.28 ± 1.27	0.84
Bicarbonate (mEq/L)	21.62 ± 2.99	22.00 ± 3.85	0.73
Total cholesterol (mg/dL)	165 (137 - 186)	157 (132 - 187)	0.80
LDL cholesterol (mg/dL)	87 (64 - 108)	80 (60 - 107)	0.68
HDL cholesterol (mg/dL)	38.0 (31.0 - 48.0)	42.5 (35.0 - 54.0)	0.03
Triglycerides (mg/dL)	175.4 ± 93.93	113.3 ± 48.26	0.0004
Albumin (g/dL)	4.01 ± 0.47	4.00 ± 0.59	0.91
Parathormone (pg/mL)	144.5 (80.5 - 344.8)	131.0 (44.0 - 320.0)	0.32
Alkaline phosphatase (U/L)	161.0 (96.25 - 217.8)	140.0 (89.0 - 205.0)	0.23
Ferritin (ng/ml)	527.0 (239.0 - 876.0)	421.0 (217.0 - 854.0)	0.67
Transferrin saturation (%)	35.09 ± 15.47	33.26 ± 10.50	0.52
Vitamin D (ng/mL)	26.69 ± 12.89	27.81 ± 15.83	0.75
TSH (mU/L)	2.0 (1.0 - 3.0)	2.0 (1.5 - 5.0)	0.02
Free T4 (mg/dL)	6.0 (1.0 - 8.0)	5.0 (1.0 - 7.3)	0.56
Aluminum (mcg/L)	14.43 ± 6.74	15.93 ± 11.0	0.42
Serology			
Anti-HCV+, n (%)	1 (1.1)	1 (11.4)	0.48
HBsAg+, n (%)	0.0 (0.0)	0.0 (0.0)	1.0
Anti-HBs+, n (%)	32 (36.0)	20 (57.1)	0.04
Anti-HIV+, n (%)	0.0 (0.0)	0.0 (0.0)	1.0

Values expressed by the mean ± SD, median (interquartile range) or by frequency; std Kt/V = standard Kt/V of weekly urea.

### Geriatric characteristics

**Figure 1:**
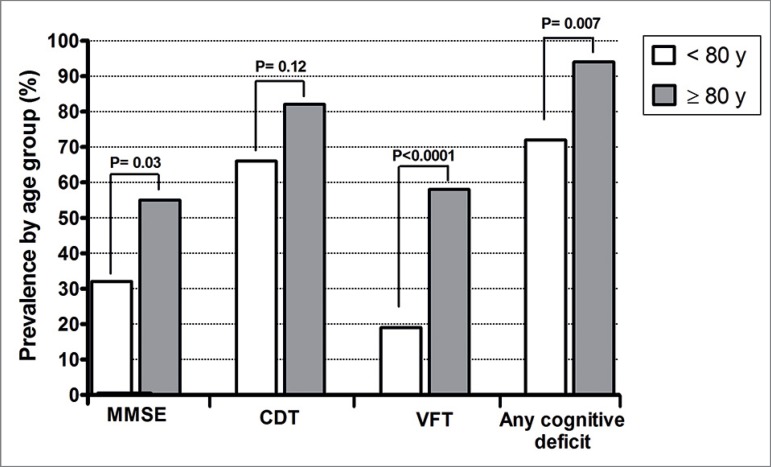
Cognitive deficit by the mini mental state examination (MMSE), clock-drawing test (CDT), verbal fluency test (VFT) and any deficit, comparing patients with < 80 years vs. ≥ 80 years.

**Figure 2:**
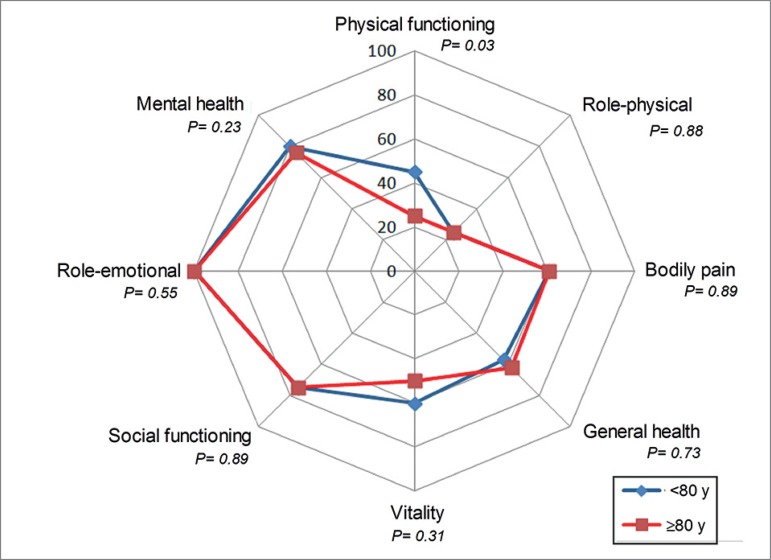
Quality of life by the SF-36, comparing patients < 80 years vs. ≥ 80 years.

Table 4 shows the main geriatric characteristics. The occurrence of at least two falls in the last year was 39.3% and 25.7% among patients with < 80 years and ≥ 80 years, respectively (*p* = 0. 20). The mean number of drugs in use was 10.4 ± 3.7, with almost all patients using polypharmacy (≥ 5 medications), and the majority with excessive polypharmacy (≥ 10 medications). Of the 124 patients evaluated, 41.9% were on benzodiazepines, and only 9.7% were on antidepressant drugs; although 38.2% presented GDS suggestive of depression. As for cognitive deficit, the very elderly had the greater impairment, both by MMSE: 54.5% versus 31.8% (*p* = 0.03), and by the VFT: 57.6% versus 19. 3% (*p* < 0.0001). According to the CDT, there was a tendency for a higher frequency of deficits among the very elderly: 81.8% versus 65.9% (*p* = 0.12). The proportion of any cognitive deficit was 93.9% among the very elderly versus 71.6% among the younger (*p* = 0.007); Figure 1. However, in a logistic regression model, the risk of the very elderly presenting any cognitive deficit lost statistical significance (*p* = 0.071) after adjustment for severe auditory deficit, coronary disease, time on dialysis and serum TSH level; Table 5. In relation to the eight domains of the SF-36 scale, there was only a difference (*p* = 0.033) in functional capacity, which was worse among the very elderly, with a median (IQR) of 25 (10 - 60) in this group versus 45 (25-69) in the < 80 years group (Figure 2).

**Table 4:** Geriatric characteristics comparing patients < 80 years vs. ≥ 80 years

Variable	< 80 years (n = 89)	≥ 80 years (n = 35)	*p* value
≥ 2 falls in the previous year (%)	35 (39.3)	9 (25.7)	0.2
Nº Medications in use	10.57 ± 3.85	9.87 ± 3.48	0.36
Polypharmacy, n (%)	85 (95.5)	34 (97.1)	1.0
Excessive polypharmacy, n (%)	51 (57.3)	18 (51.4)	0.69
Benzodiazepine use, n (%)	38 (42.7)	14 (40.0)	0.84
Antidepressant use, n (%)	9 (10.1)	3 (8.6)	1.0
Cognitive deficit, n (%)*			
MMSE	28 (31.8)	18 (54.5)	0.03
CDT	58 (65.9)	27 (81.8)	0.12
VFT	17 (19.3)	19 (57.6)	< 0.0001
Any cognitive deficit	63 (71.6)	31 (93.9)	0.007
Depression (GDS≥6), n (%)	33 (37.1)	14 (41.2)	0.68
Quality of life (SF-36)			
Functional capacity	45 (25 - 69)	25 (10 - 60)	0.03
Disabilities	25 (0 - 50)	50 (25 - 100)	0.88
Pain	61 (31 - 100)	61 (22 - 100)	0.89
General health status	57 (40 - 72)	62 (30 - 90)	0.73
Vitality	60 (45 - 80)	50 (32.5 - 78)	0.31
Social aspects	75 (38 - 100)	75 (25 - 100)	0.89
Emotional aspects	100 (33 - 100)	100 (100 - 100)	0.55
Mental health	80 (64 - 92)	76 (56 - 88)	0.23

Values expressed by the mean ± SD, median (interquartile range), or by frequency. Polypharmacy: ≥ 5 drugs; excessive polypharmacy: ≥ 10 medicines; MMSE = mini mental status exam; CDT = clock drawing test; VFT = verbal fluency test; GDS = geriatric depression scale; SF-36 = health-related quality-of-life questionnaire Short Form (SF)-36

* n of patients submitted to the cognitive tests was 88 (< 80 years) and 33 (≥ 80 years).

**Table 5:** Analysis of the variables associated with any cognitive deficit (MMSE, CDT or VFT) in the logistic regression model

Variable	Odds ratio	CI 95%	*p* value
Age ≥ 80 years	3.59	(0.90 - 14.40)	0.071
Coronary disease	0.47	(0.11 - 2.08)	0.32
Severe hearing deficit	4.37	(0.74 - 25.92)	0.104
TSH (um/L)	0.99	(0.72 - 1.35)	0.94
Time in dialysis (months)	0.99	(0.97 - 1.01)	0.32

CI 95% = 95% confidence interval; MMSE = Mini mental state exam; CDT = clock drawing test; VFT = verbal fluency test.

## Discussion

The elderly with more than 80 years of age is the portion of the population that has been growing the most in the world and, more rapidly, in Brazil. Consequently, the incidence of CKD is also increasing in its various stages at this extreme of age, but studies analyzing this population separately are scarce. In the present study, with chronic hemodialysis patients, we found that the elderly are more cognitively impaired than than oldest old (less than 80 years of age), but we did not find differences between the two groups in other relevant clinical aspects of elderly health. To our knowledge, this is the first study to specifically compare very elderly patients with those lesser elderly on hemodialysis in relation to the main geriatric syndromes.

Regarding mood disorders, we found a high prevalence of depression, with no difference involving those older than 80 years. There is evidence that depression is a predictor of mortality in this population.^18^ To screen for depression, we used the GDS, which has validation for the Brazilian elderly, using the cutoff point of 5/6 (no case/case). Paradela et al., in a study with elderly patients in a general outpatient clinic in the city of Rio de Janeiro, demonstrated sensitivity of 81% and specificity of 71% with this same cut-off point.^7^ In the USA, the GDS validated for elderly patients on hemodialysis, had the most accurate cutoff point 4/5, with sensitivity of 63% and specificity of 82%, and a prevalence of depression of 32.3% was found in that population.^19^ However, in the present study, we found that the items 8 and 14 of this questionnaire, "Do you think your situation has no way out?" And "Do you think your situation is hopeless?" were often answered negatively simply because the participant was aware of the disease's prognosis and the irreversible loss of renal function without manifesting depressive signs. Future studies to validate the Brazilian version of GDS for elderly on hemodialysis are still necessary.

It was clear that most of the depression cases diagnosed in the study had no prior diagnosis of this condition. In parallel to the low attention given to mood disorders, with little use of antidepressants or other treatments directed to the cause of anxiety symptoms, many patients were in prolonged and unsupervised use of benzodiazepine. Benzodiazepine is a class of medications of high risk for this population, recognized among the main drugs potentially inappropriate for use in the elderly,^20^ because they have a well-documented association with falls and fractures,^21^ mortality secondary to fracture^22^ and cognitive impairment.^23^

Elderly patients undergo hemodialysis due to associated multi-morbidities, but even though it is not possible to substantially reduce the number of drugs in use, it is essential to ensure that each patient receives only appropriate, effective, safe and convenient medicines. This will only be possible with a careful and routine review of the drugs being used, which probably does not occur in the current model of hemodialysis patient care.^24^

One possible consequence of polypharmacy is the high rate of falls and hospitalizations in the study population. More than a third of patients had had at least two falls in the past year. Most of these falls had not even been reported to the healthcare professionals at the dialysis clinic, with prevention measures often being neglected. The hospitalization rate we found was lower than those described in the literature,^1^ but this probably reflects underreporting, since the electronic medical records used in the participating clinics do not usually have information regarding adverse events that did not generate hospitalization or care in emergency services. Thus, we chose to use the information obtained through the patient's or family's report, retrospectively, at the time of this study, with memory bias.

As for cognitive tests, we chose to use the MMSE, the VFT and the CDT. Thus, we were able to evaluate several domains of cognition: orientation in time and space, memory, attention, calculation, language, visuospatial abilities, executive function and abstract thinking.

For the MMSE, we used the cut-off points suggested by Almeida et al.,^16^ who demonstrated sensitivity and specificity for the diagnosis of dementia of 80.0% and 71.0% for patients without schooling, and 77.8% and 75.4%, for patients with previous formal education, respectively. Through this cutoff point, we found a higher prevalence of cognitive deficit among the very elderly. This finding corroborates what was expected, by analogy from the general population, despite the possible existence of some protective biases among octogenarian and nonagenarians, which, in our analysis, had a lower prevalence of comorbidities known to be associated with the risk of dementia.

The VFT category, which was used by us, has higher sensitivity in the detection of Alzheimer's disease, and reflects the temporal lobe's ability to retrieve information stored in memory through the organization of thought and word search strategies.^25^ It has high accuracy for the diagnosis of dementia in the elderly and, due to the practicality of its application, it is recommended its association with the MMSE in the screening of this geriatric syndrome.^26^ In the evaluation by the VFT we also show a higher prevalence of cognitive deficit among the very elderly compared to the less elderly.

On the other hand, the CDT was the only test in which we did not find a difference between very old patients and those less than 80 years-old. This result may be related to the higher prevalence of diabetes among less elderly patients, since CDT is a test that better evaluates the executive function, and the neurodegenerative process affecting hemodialysis patients may have more influence of cerebrovascular factors secondary to diabetes, with a greater prevalence of multi-infarct dementia, which has a greater impact on this cognitive domain.^27^ The CDT has different scoring methods, and we used the one recommended by Manos and Wu.^11^ However, although previously translated and adapted for the Brazilian elderly,^14^ there is evidence that CDT is not a valid test for populations with low schooling,^28^ which corresponds to a good part of our sample. Fusikawa et al. found good reliability of the test even in a Brazilian population with low schooling,^29^ but they used the Schulman method of application, and there are still a lack of studies that reproduce the same result, determining consistency in similar populations. Lourenço et al. suggest that both methods have similar accuracy for dementia screening in the elderly;^28^ thus, a future study comparing the results obtained by different methods in this population would be interesting.

When we considered the presence of any cognitive deficit, its prevalence was higher among individuals aged 80 years or older. However, after adjustment for variables that were different between groups and could influence cognition, the association between being too old and having some cognitive deficit was attenuated.

For the quality-of-life assessment, we used the SF-36, although there is a more specific instrument for the dialysis population, the Kidney Disease Quality of Life Short Form (KDQOL-SF), also validated for Portuguese.^30^ Our option for SF -36 was because it is a widely used instrument, even for patients on dialysis, and that, unlike KDQOL-SF, has already been validated for the elderly,^31^ but we believe that this may be a limitation of our study.

Another limitation is that we evaluated only those patients in hemodialysis for more than 3 months, but many may not survive the first few months of treatment, and because the analysis involved prevalent instead of incident patients, there would be a survival bias. In addition, the very elderly with stage 5 CKD are likely to experience a positive clinical selection bias when they are referred to the commencement of RRT.

The current model of care for dialysis patient is focused on the control of CKD and its complications, such as hypervolemia, electrolytic disturbances, anemia and bone disease. However, the elderly with ESRD have many comorbidities and geriatric syndromes that cannot be modified by dialysis, with a direct impact on survival, cognition and quality of life. Having biochemical parameters as a priority goal and instructing to seek a general hospital in case of clinical complications translates into a high financial cost medicine, with questionable value for patients. In this population, a comprehensive geriatric assessment and a holistic clinical follow-up, focused on the well-being, prevention of complications and palliative care associated with dialysis treatment, are relevant. All such care should be incorporated into the dialysis unit routines, which are the "second home" of patients, due to the time spent weekly with dialysis therapy. In addition to optimizing patients' time and increasing adherence to clinical follow-up and treatment, the use of the dialysis unit as a place to centralize all this care would facilitate communication and interaction among professionals involved in care.

Regarding the quality-of-life assessment, SF-36 domains related to tphysical limitations, pain, general health, vitality, social aspects, social limitations and mental health were similar between younger and older adults. Nevertheless, the dimension related to functional capacity was worse among the very old, which probably reflects the greater dependence of these individuals on day-to-day activities and could be better investigated by future studies, through instruments for the evaluation of functionality.

In conclusion, the results of this study showed that oldest old patients on chronic hemodialysis have a high prevalence of cognitive deficit, which is even higher among the very elderly. Quality of life was not worse among elderly, except for the aspect of functional capacity.
